# Integrating Health in the Water-Energy-Food Nexus: A Comprehensive Review of Interdependencies, Challenges, and Future Research Opportunities

**DOI:** 10.1007/s40572-025-00516-4

**Published:** 2025-12-10

**Authors:** Rashed Albatayneh, Rabi Mohtar, Zainab Ashkanani, Bassel Daher, Wael K. Al-Delaimy

**Affiliations:** 1 Zachry Department of Civil and Environmental Engineering, Texas A&M University, College Station, TX USA; 2 Department of Biological and Agricultural Engineering, Texas A&M University, College Station, TX USA; 3 Texas A&M Energy Institute, Texas A&M University, College Station, TX USA; 4 Herbert Wertheim School of Public Health, University of California San Diego, San Diego, CA USA

**Keywords:** Water-energy-food-health nexus, Public health, Risk assessment, System engineering, Resilience

## Abstract

**Purpose of Review:**

The Water-Energy-Food (WEF) Nexus has become a crucial framework for understanding resource interdependencies, nevertheless its integration with health remains underexplored. This review paper examines the current literature in the Water Energy Food Health (WEFH) Nexus, emphasizing the complex relationships between resource security and public health outcomes. Through a systematic literature review, the existing research trends, methodological approaches, and regional disparities in WEFH studies are analyzed.

**Recent Findings:**

While health is an inherent component of resource systems, it is often treated as an externality rather than a central determinant. The review revealed that out of 1175 research articles screened, only 21 discussed WEFH as an integrated nexus. Hence, there is a crucial need for a comprehensive Water, Energy, Food, and Health research.

**Summary:**

The paper proposes a conceptual WEFH integration that positions health as both an outcome and a driver of sustainable resource management based on epidemiological evidence. By analyzing health metrics implicit to the WEF nexus, the study provides insights for researchers and policymakers seeking to develop holistic strategies for resilience and equity in resource governance.

## Introduction

The Water-Energy-Food (WEF) nexus has emerged as a critical framework for understanding the complex interdependencies that govern resource security and sustainable development in the 21st century. Based upon systems thinking, the nexus paradigm emphasizes the synergies and trade-offs in managing water, energy, and food systems: resources that are inter-linked yet often siloed in policy and academic dialogue. However, while the WEF nexus has advanced interdisciplinary research, its conceptualization has historically underprioritized health, a dimension that is both a determinant and an outcome of resource accessibility, equity, and sustainability. This oversight is particularly critical given that water, energy, and food systems are foundational pillars of public health. As global crises, from climate change and conflicts to pandemics, intensify the pressure on these systems, the necessity to integrate health into the WEF nexus (hereafter termed the Water Energy Food Health, or WEFH nexus) has never been more urgent. This framework acknowledges that the interactions between these sectors are complex and multidimensional, influencing each other through direct and indirect pathways [1, 2]. This integration represents not merely an academic exercise but a practical necessity for developing resilient systems that protect human wellbeing while ensuring sustainable resource management.

Integrating health into the WEF nexus is not simply an additional layer of complexity, it is a fundamental shift that recognizes health as both an outcome and a determinant of sustainable resource management. Health is a critical indicator of the resilience and functionality of water, energy, and food systems. This is especially true in low resource countries and underserved populations. For instance, access to clean water and adequate sanitation directly impacts disease prevalence and energy poverty exacerbates adequate access to resources. Similarly, food security is directly tied to malnutrition and long-term immunological disorders, making the inclusion of health in the resource nexus essential for addressing systemic inequities [3]. The integration of health metrics into WEF frameworks enables more accurate assessment of policy trade-offs by revealing how interventions in one sector can generate favorable or adverse consequences in others and, ultimately, impact human wellbeing. Despite its critical importance, health integration into the WEF nexus faces significant challenges. Fragmented governance structures, disciplinary and data silos, and methodological barriers limit comprehensive approaches that capture the complex interactions between resource systems and health outcomes. These challenges are particularly pronounced in regions that face compounded resource insecurities and health disparities.

The primary objectives of this paper are to (i) synthesize the current state of WEFH nexus research and provide a comprehensive analysis of the interdependencies within the nexus and their implications for public health; (ii) identify key trends in WEFH research by highlighting methodological and regional gaps, outline future opportunities to enhance the framework’s effectiveness; (iii) conceptualize and analyze how interactions among water, energy, and food influence public health in a wholistic framework.

By addressing these dimensions, the paper seeks to advance understanding of the WEFH nexus and provide actionable insights for fostering sustainable, equitable, health-focused resource management strategies. This paper is organized into four main sections to provide a comprehensive exploration of the WEFH nexus. The introduction outlines the importance of integrating health into the WEF nexus, establishing the context and objectives of the review. The second section presents the methodology of literature search and the outputs. The third section examines trends and focus areas in existing literature to provide insights into methodological approaches, geographic coverage, and thematic priorities. The final section identifies research gaps, discusses limitations, and proposes future directions that integrate health more effectively into the WEFH nexus. Together, these sections offer a structured roadmap to advance understanding, research, and policy development in this critical interdisciplinary field.

## Methodology: Systematic Literature Survey

A systematic literature survey was conducted to explore the interdependencies within the Water-Energy-Food-Health (WEFH) nexus and its implications for public health. Various review methodologies have been presented in literature. Muhirwa et al., 2022, presented a systematic methodology leading to critically analyze WEF studies and official project reports in Africa, with similar other research articles [4–7]. This article review process involved selecting appropriate academic databases, formulating comprehensive search queries, applying inclusion and exclusion criteria, and systematically organizing the extracted data, as detailed in the following sections.

### Database Selection and Search Strategy

The search was performed using Web of Science and PubMed, selected for their extensive coverage of interdisciplinary topics, including environmental science, public health, engineering, and policy research. Keywords and their synonyms were used across the four primary nexus components of water, energy, food, and health to search titles and abstracts. Boolean operators (e.g., AND, OR) were applied to refine search queries and ensure inclusion of diverse perspectives and interdisciplinary research contributions. Multiple rounds of searches were conducted using different combinations of terms to capture the breadth of research at various intersections of the WEFH nexus. The specific search strings and the number of retrieved articles are summarized in Table 1. It must be noted that the term “FEW” was not included as it outputs imprecise results. However, the inclusion of “Water OR Energy OR Food” is expected to cover all missed articles.

**Table 1:** Search strings and results for WEFH literature survey

Search String	Date of Search	Year Filter	Database	Papers Found
(“Water Energy Food Health nexus” OR “WEFH” OR “WEF nexus”) AND (health OR “public health” OR disease OR “health outcomes”)	3/18/2025	2010-now	Web of Science	22
		2010-now	PubMed	24
(water AND energy AND food) AND (health OR “public health”) AND (integration OR interdependence OR interaction)	10/9/2024	2010-now	Web of Science	169
		2010-now	PubMed	39
(water OR energy OR food) AND health AND (nexus OR integration OR interdependence) AND (disease OR epidemiology)	1/6/2025	2010-now	Web of Science	810
		2010-now	PubMed	477
(“WEF nexus”) AND (health OR “public health”) AND (methodology OR framework OR model)	10/17/2014	2010-now	Web of Science	10
		2010-now	PubMed	2
(“Water-Energy-Food nexus”) AND health AND (impacts OR effects OR outcomes)	10/17/2014	2010-now	Web of Science	4
		2010-now	PubMed	5
(“WEF nexus”) AND health AND (policy OR governance OR “resource management”)	10/17/2024	2010-now	Web of Science	15
		2010-now	PubMed	2
(engineering AND health) AND (“WEF nexus” OR “Water-Energy-Food nexus”) AND (methodology OR framework)	10/17/2024	2010-now	Web of Science	0
		2010-now	PubMed	0
(“WEF nexus”) AND (malnutrition OR “waterborne diseases” OR “infectious diseases”)	10/18/2024	2010-now	Web of Science	2
		2010-now	PubMed	1
(water AND energy AND food) AND (“public health”) AND (“developing countries” OR “low-income countries”)	10/18/2024	2010-now	Web of Science	11
		2010-now	PubMed	16
(“WEF nexus”) AND health AND (“research gaps” OR “future research” OR “knowledge gaps”)	10/26/2024	2010-now	Web of Science	2
		2010-now	PubMed	1

### Inclusion and Exclusion Criteria

Predefined inclusion and exclusion criteria were applied to ensure the relevance and quality of the selected studies. Studies are included if they (i) were published in peer-reviewed journals, (ii) focused on at least one aspect of the WEF nexus and health interdependencies, (iii) addressed public health outcomes within the nexus framework, and (iv) were published within the last 15 years (2010–present) to capture recent advancements. Conversely, studies were excluded if they (i) lacked a clear connection to health within the nexus, (ii) did not adopt a nexus-based perspective, or (iii) were non-peer-reviewed.

### Selection Process and Data Extraction

**Fig. 1:**
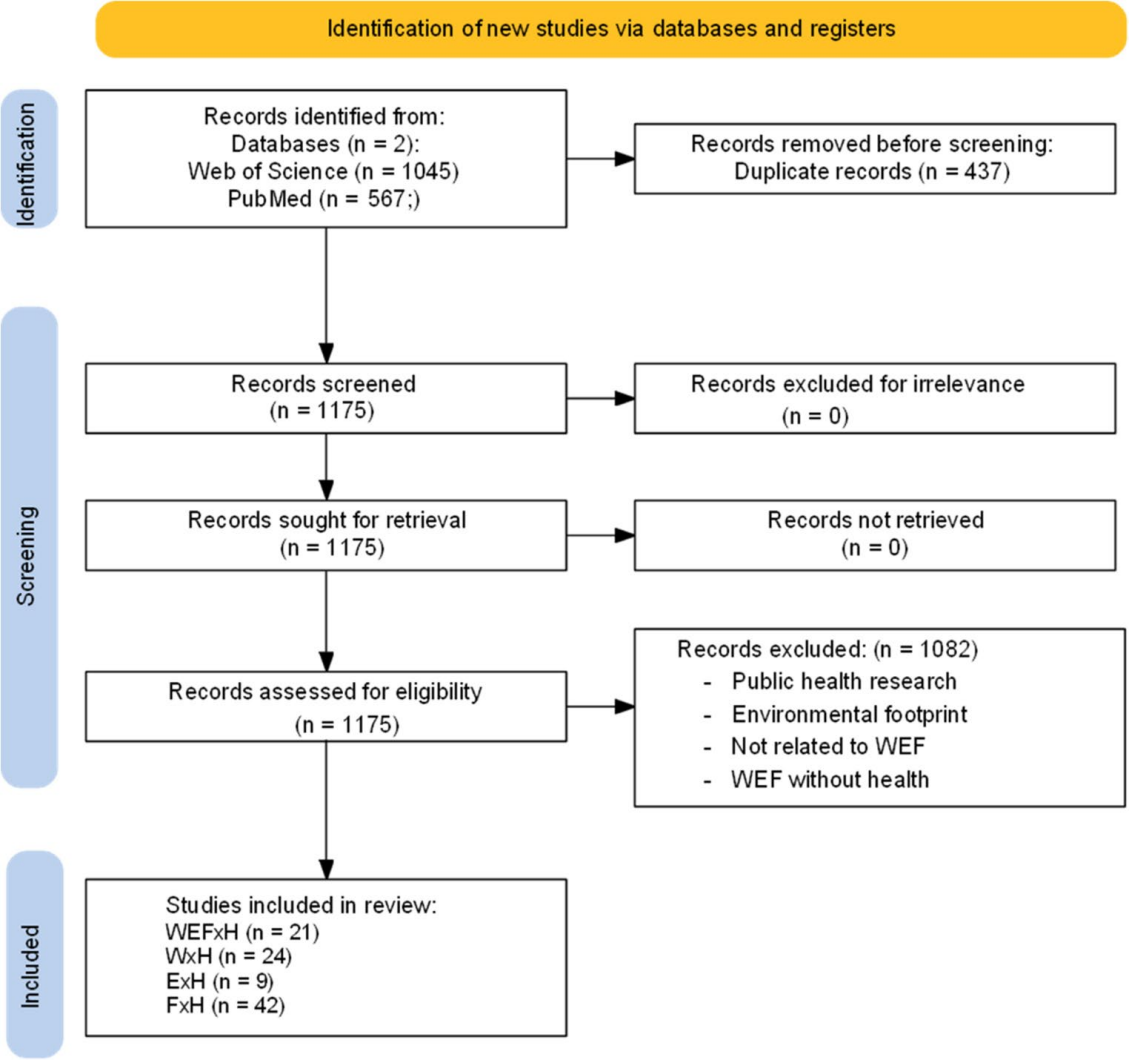
PRISMA flow chart for WEFH literature survey

The search and selection process was conducted in multiple stages. Initially, search queries were applied to retrieve a wide range of potentially relevant studies. These articles were evaluated for relevance following the Preferred Reporting Items for Systematic Reviews and Meta- Analyses (PRISMA) guidelines as shown in Fig. 1 [8]. Titles and abstracts were screened to assess their alignment within the WEFH nexus and the presence of keywords. Articles that passed this initial screening underwent a full-text review to evaluate their contribution to the study’s objectives. This multi-step selection process ensured the inclusion of studies that provide meaningful insight into the nexus framework and its public health implications.

With studies collected and screened, key information was systematically extracted, focusing on thematic areas, research focus, geographic scope, methodological approaches, policy implications, and health-related outcomes within the nexus. The extracted data were organized into a structured database to facilitate literature mapping, trend analysis, and gap identification. This systematic approach enabled a comprehensive synthesis of existing research, providing a foundation for assessing the interconnections between water, energy, food, and health.

### Study Selection Summary

Figure 1 presents the PRISMA flow diagram summarizing our systematic review process. Of the 1,610 records initially identified, 103 publications met all inclusion criteria after removing duplicates and screening abstracts and full texts. The primary reasons for exclusion were lack of explicit health-nexus connections (37%), focus on single resources without nexus perspectives (26%), and theoretical papers lacking substantive analysis (14%).

## Results and Analysis

### Bibliometric Analysis of Research Trends

A bibliometric analysis conducted on the included research papers published to examine thematic trends and conceptual linkages within the WEFH literature. Using VOSviewer software [9], terms extracted from titles and abstracts resulted in 4503 initial terms. After applying a minimum occurrence threshold of 8 and excluding general terms, 38 key terms were identified and analyzed in a co-occurrence network. This threshold was determined based upon author judgment after a few trials to get highest frequency terms without compromising quality.

**Fig. 2:**
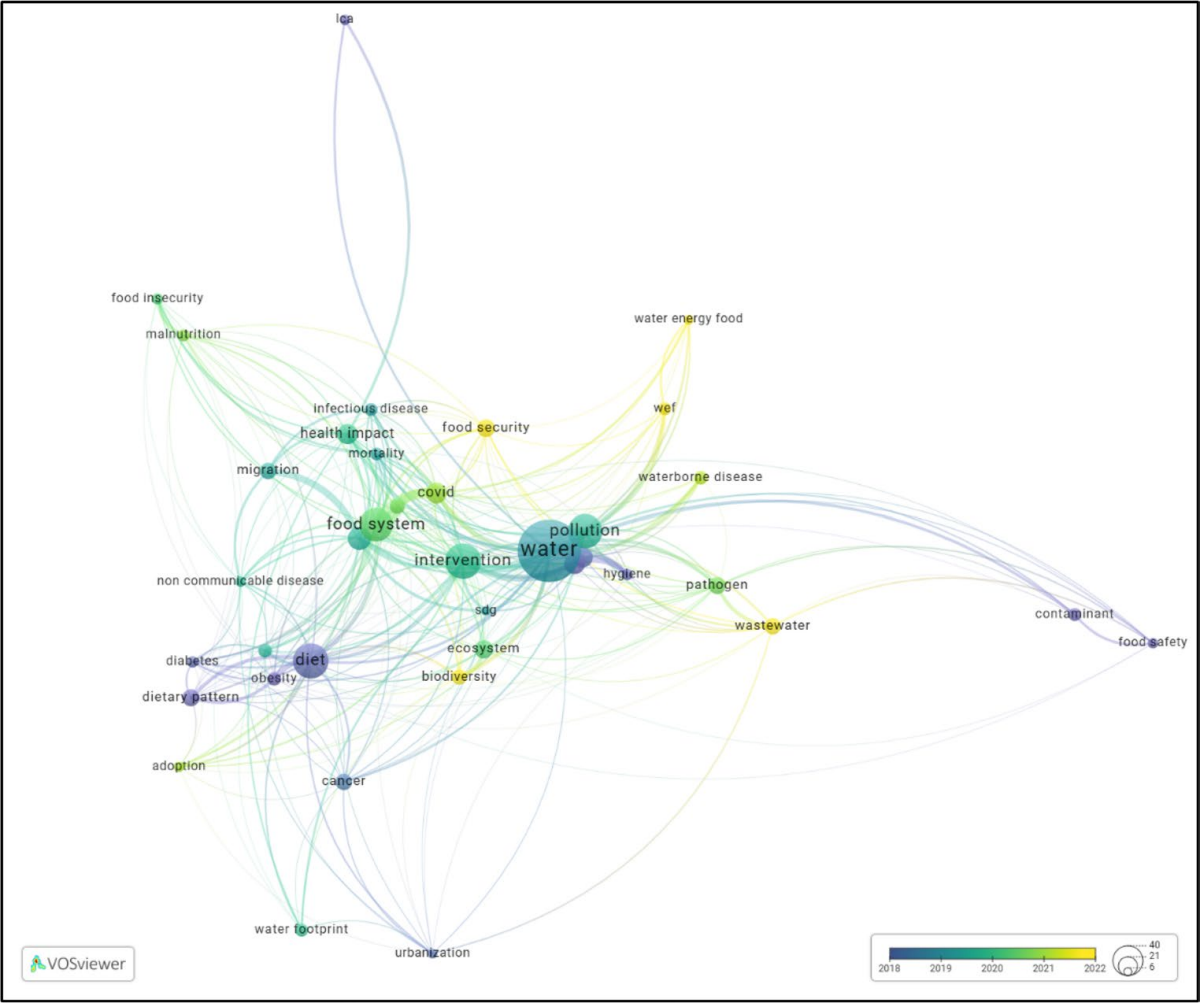
The co-occurrence network of key terms in the WEFH nexus literature

The co-occurrence network in Fig. 2 visualizes those terms most often interconnected and provides insights into the current state of WEFH nexus research. The network reveals five distinct clusters representing major research themes: (1) water resource management and contamination, (2) food systems and nutrition, (3) energy production and climate impacts, (4) health outcomes and disease burden, and (5) governance and policy integration. Analysis showed the term “Water” emerged as the most central node in the network, serving as a connection point between environmental contamination, food systems, and health-related nodes like “non-communicable diseases”. Notably, the analysis highlighted significant disparities in research focus. While water-health connections dominated the literature, energy-health linkages received comparatively less attention, a critical gap given the substantial health impacts of energy production, distribution, and consumption through air pollution, occupational hazards, and climate effects.

As shown above, the literature survey resulted in 21 research papers focusing on health within the WEF nexus. These papers are presented and summarized in Table 2.

**Table 2:** Summary of papers screened focusing on WEF and H interaction

Authors and Year	Title	Abstract Summary	Main Findings	Region	Reference
(Javan et al., 2025)	Interrelated issues within the Water-Energy-Food nexus with a focus on environmental pollution for sustainable development: A review	Examines pollution within the WEF nexus, focusing on pollution sources, interconnectedness, and feedback loops to inform sustainable policy development.	Identifies pollution sources and their interconnected impacts across the WEF nexus, highlights complex feedback mechanisms, emphasizes the need for integrated cross-sector policies.	Global	[4]
(Yeboah SIK et al., 2024)	Water, Environment, and Health Nexus: Understanding Risk Factors in Tano River Basin	Investigates waterborne disease drivers and risks in Ghana’s Tano River Basin.	Poor sanitation, hygiene, and illegal mining drive diseases; flood-prone areas exacerbate risks.	Ghana	[10]
(Kim et al., 2024)	Role of Biodiversity in Climate, Food, Water, Energy, Transport, and Health Nexus	Explores biodiversity’s interconnections with climate, food, water, and other sectors in Europe.	Highlights biodiversity’s critical role in sustaining ecosystems and influencing policy for nexus interlinkages.	Europe	[11]
(Rhouma et al., 2024)	Trends in the Water-Energy-Food Nexus Research	Analyzes global WEF nexus research trends through bibliometric analysis.	The WEF nexus is under-researched in agriculture; integrating health adds a crucial dimension for sustainable development.	Global	[5]
(Mutanga et al., 2024)	Implementation of Water-Energy-Food-Health Nexus in a Climate-Constrained World	Reviews the integration of health into the WEF nexus framework for South Africa in light of climate challenges.	Health inclusion in the WEF nexus can guide sustainable development and policy alignment for resource management in South Africa.	South Africa	[1]
(Salahodjaev & Sadikov, 2024)	Renewable Energy, CO2 Emissions, and Economic Factors in High CHD Mortality Countries	Explores the interplay between renewable energy, CO2 emissions, and economic factors in countries with high coronary heart disease rates.	Renewable energy adoption reduces CO2 emissions and improves environmental quality, highlighting policy needs for sustainable energy in vulnerable regions.	Global	[12]
(Botai et al., 2024)	Assessment of Rural Livelihoods, Health, and Wellbeing in South Africa and Kenya	Assesses rural livelihoods using the WEF nexus framework in South Africa’s Vhembe District and Kenya’s Narok County.	Sustainable livelihood indicators reveal more sustainability in Vhembe than Narok, emphasizing the need for targeted policy interventions.	South Africa, Kenya	[13]
(Javan et al., 2024)	A Review of Interconnected Challenges in the WEF Nexus: Urban Pollution Perspective	Reviews pollution challenges within the WEF nexus, focusing on urban environments and sustainable practices.	Highlights urban pollution as a major challenge in the WEF nexus, advocating for integrated policies and the use of advanced technologies for mitigation.	Global	[14]
(Urbinatti et al., 2023)	Nexus Narratives in Urban Vulnerable Places	Evaluates the role of municipal health programs in addressing WEF nexus challenges in urban Brazil.	Municipal health programs indirectly incorporate WEF nexus principles, promoting sustainability and resilience in vulnerable urban areas.	Brazil	[15]
(Rapinski et al., 2023)	Local Food Systems under Global Influence	Analyzes how globalization impacts local food systems, nutrition, and environmental health in five diverse socio-ecosystems.	Globalization induces nutritional and health transitions, necessitating strategies to maintain sustainability and traditional food systems.	Canada, Senegal, Guadeloupe	[16]
(Lund et al., 2022)	Tracing the Inclusion of Health in Food-Energy-Water Nexus in Senegal River Basin	Examines health impacts of dam projects and their integration into nexus frameworks in the Senegal River Basin.	Health is critical but underrepresented in dam management; emphasizes integrating health without compromising resources.	Senegal River Basin	[17]
(Nuwayhid & Mohtar, 2022)	The WEF Nexus: Health as Yet Another Resource	Advocates for incorporating health into the WEF nexus framework for more comprehensive solutions.	Health is underexplored in WEF nexus studies, requiring integration to address human well-being effectively.	Global	[2]
(Al-Saidi & Hussein, 2021)	The WEF Nexus and COVID-19: Towards a Systematization of Impacts	Evaluates COVID-19’s impacts on the WEF nexus and response strategies.	Highlights challenges in WEF resource management, emphasizing localization and adaptation strategies post-COVID.	Middle East	[18]
(Hirwa et al., 2021)	Insights on Water and Climate Change in the Greater Horn of Africa	Examines the WEF-Biodiversity-Health nexus in the Greater Horn of Africa through water footprints and virtual water analysis.	Highlights the unsustainable water footprints of staple crops and the necessity of integrated policies for WEF nexus and biodiversity conservation.	Greater Horn of Africa	[19]
(Calder RSD et al., 2021)	COVID-19 Reveals Vulnerabilities of the Food-Energy-Water Nexus to Viral Pandemics	Evaluates how the COVID-19 pandemic impacted the interconnectedness of the FEW nexus and exposed vulnerabilities.	Identifies critical trade-offs and feedbacks between FEW systems during pandemics, emphasizing the need for robust health-integrated nexus frameworks.	Global	[20]
(Mabhaudhi et al., 2019)	The WEF Nexus as a Tool to Transform Rural Livelihoods in Southern Africa	Discusses WEF nexus applications to improve rural livelihoods and health in Southern Africa.	Integrated WEF management enhances resilience and development, addressing imbalances in resource allocation.	Southern Africa	[3]
(Song, Han, et al., 2019)	Potential Water-Food-Health Nexus in Urban China	Examines dietary changes’ impact on the water-food-health nexus in urban China.	Urbanization drives water-intensive diets, increasing health risks; calls for healthier dietary choices for sustainability.	China	[21]
(Song, Gao, et al., 2019)	Shift from Feeding to Sustainably Nourishing Urban China	Analyzes dietary shifts in urban China and their implications for health and environmental sustainability.	Urban diets high in animal products lead to environmental degradation and health risks, urging policies for sustainable and healthy nutrition.	China	[22]
(J. Finley et al., 2019)	Understanding the Intersection of Climate Change, Health, Agriculture, and Nutrition	Explores the links between climate change, agriculture, and nutrition using type 2 diabetes as a case study.	Emphasizes the role of team science to address nutritional and health challenges amid changing environmental and food systems.	Global	[23]
(Raiten & Aimone, 2017)	The Intersection of Climate, Food, Nutrition, and Health	Discusses the interconnected impacts of climate/environmental change on food systems, nutrition, and health.	Highlights the need for integrated approaches to address climate change impacts on nutrition and health, especially in vulnerable populations.	Global	[24]
(Islas-Espinoza & de las Heras, 2015)	WEF Nexus: From Cancer Effects of Xenobiotics to Integrated Sustainable Technologies	Examines the impact of xenobiotics from WEF technologies on health, focusing on sustainable solutions.	Current WEF technologies contribute to health risks, including cancers, emphasizing need for integrated, non-toxic systems.	Global	[25]

### Temporal and Regional Analysis of WEFH Research

**Fig. 3:**
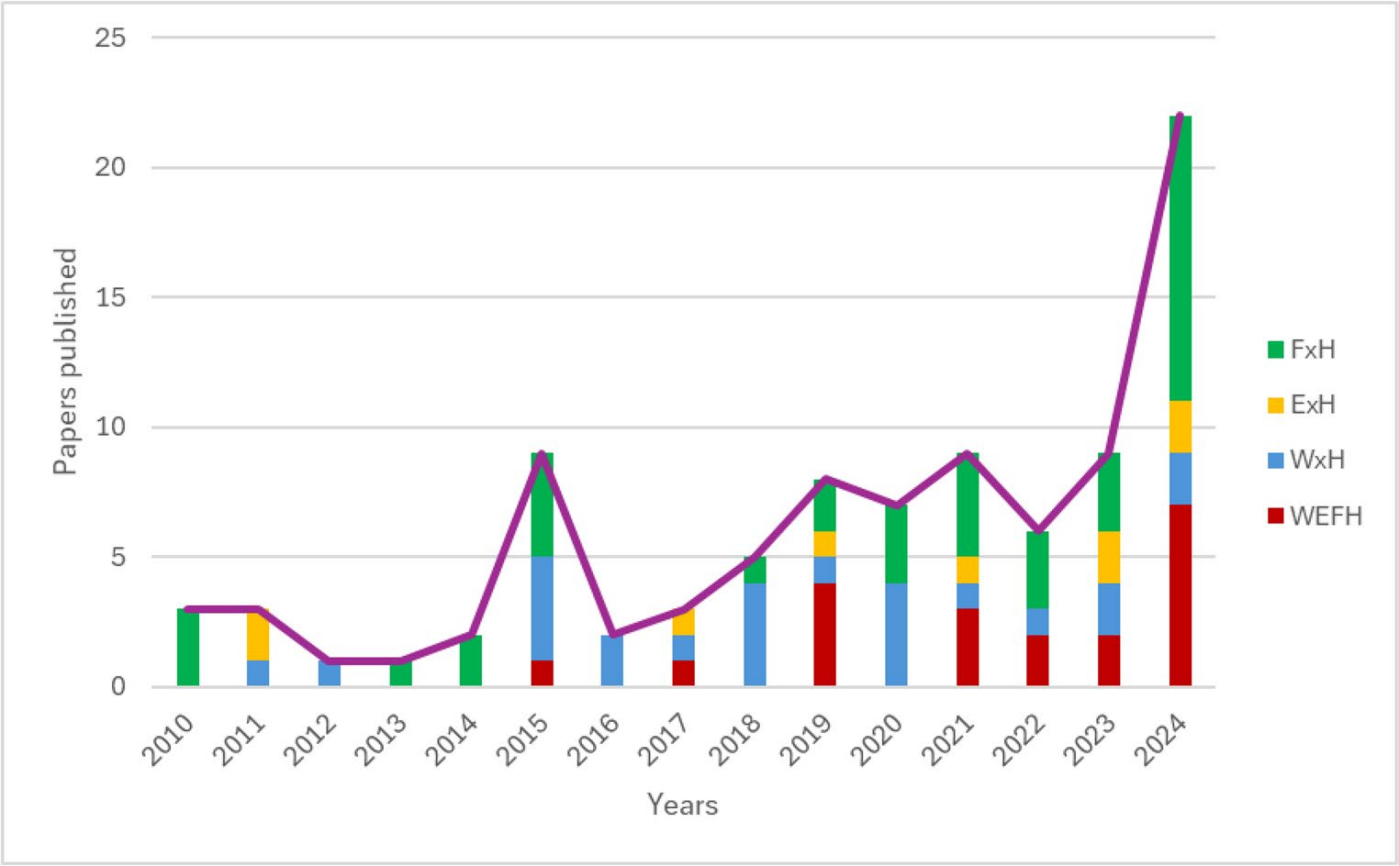
Global interest of WEFH sectors along recent 15 years

Figure 3 illustrates the temporal trend in WEFH publications from 2010 to 2025. The data reveals three distinct phases in research development: emergence (2010–2015) with sparse publications, growth (2016–2022) showing steady increase corresponding with rising global attention to systems thinking and the Sustainable Development Goals (SDGs), acceleration (2023–2025) marked by rapid expansion of the field. This pattern suggests growing recognition of health’s importance in shifting toward holistic approaches, likely catalyzed by global events such as the COVID-19 pandemic and intensifying climate impacts on resources and health. Despite recent increases in publications, the overall research output remains limited relative to the global importance of the nexus. Even in peak years, studies integrating WEFH remain few, indicating a need for greater institutional support, funding, and interdisciplinary collaboration to advance the field.

**Fig. 4:**
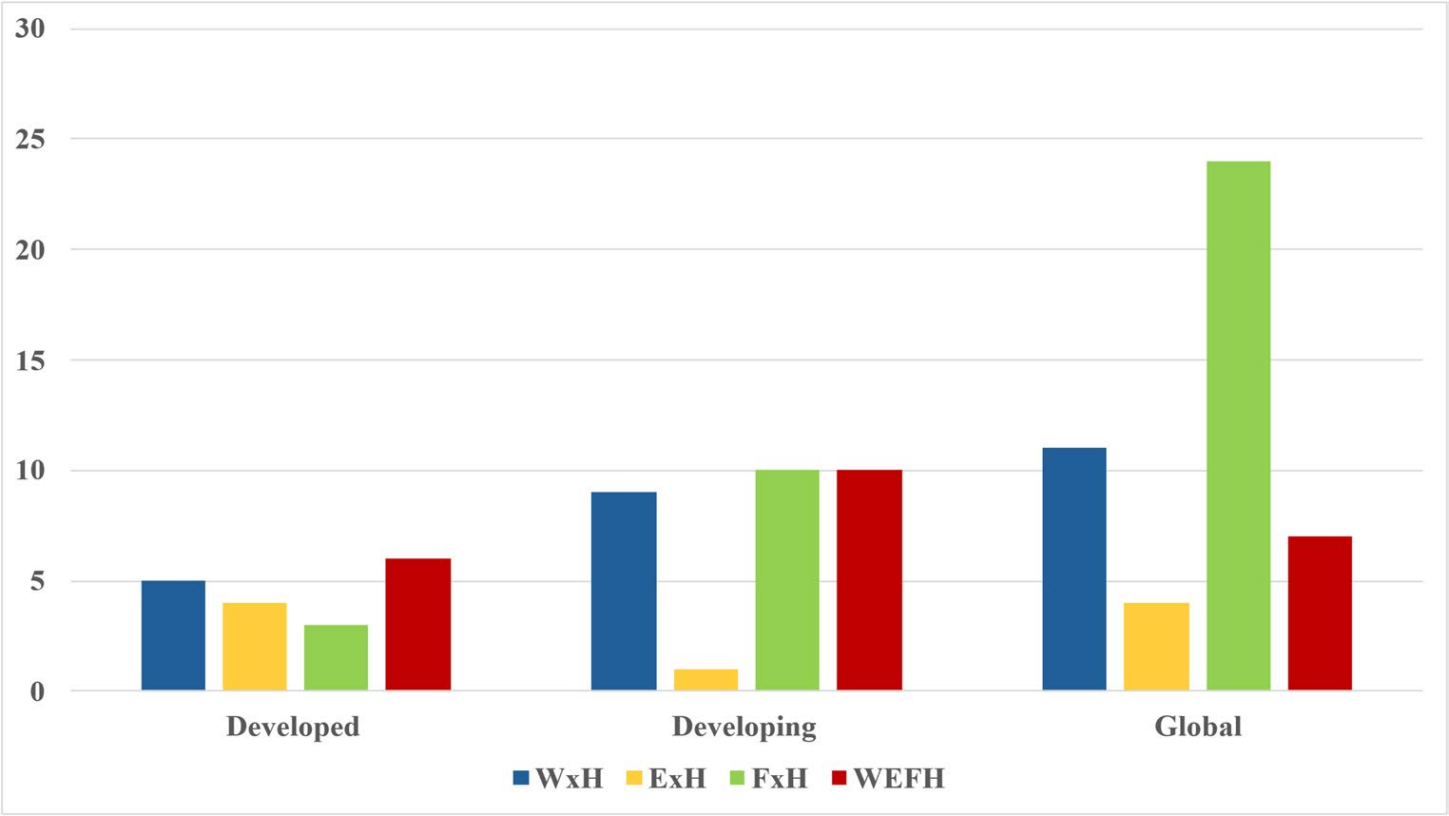
Regions Researched in Literature

Figure 4 shows the distribution of research categorized based upon regional development [26]. Analysis shows relatively balanced research sectors in developed regions, while energy-health (ExH) receives less attention in developing regions. The integrated WEFH nexus is moderately explored across all regions, with less prominent presence in global research. Notably, food-health (FxH) dominates global studies, while energy-health (ExH) remains underexplored across all contexts, highlighting a potential research gap in the role of energy within the WEFH nexus.

This regional analysis reveals concerning disparities in research focus. Low- and middle-income regions, where resource insecurities and health burdens are often most severe, remain underrepresented in the literature. This disparity can be largely attributed to two key constraints: the lack of detailed, disaggregated data in developing regions and limited funding for interdisciplinary research. Funding streams in these regions often prioritize sector-specific projects, limiting opportunities for integrated WEFH research. This gap is particularly problematic given that these regions face the greatest challenges in balancing resource security with health protection. The limited research in these contexts restricts development of appropriate policies and interventions tailored to local needs and constraints.

### Methodological Approaches in WEFH Research

A range of methodologies are used to study the WEFH nexus, with most studies employing a mixed-methods approach. Three predominant methodological categories emerged from our analysis.

**Quantitative Methods** are common, with statistical analysis, econometric modeling, and spatial analysis used to quantify WEFH components relationships and assess health impacts, livelihoods, and the environment [1, 3, 10–12, 17, 21, 24]. These approaches provide empirical evidence of nexus relationships but often struggle to capture complex feedback dynamics and social dimensions.

**Qualitative Methods**, such as literature reviews, narrative analysis, and interviews, are used to explore the social, political, and cultural dimensions of the nexus and to understand stakeholder perspectives [2, 13–16, 18–20, 25, 27]. These studies provide rich contextual understanding but may lack generalizable insights needed for broader policy application.

**Systems Modeling and Simulation Techniques** are increasingly used to assess the WEFH nexus and its impacts on health, livelihoods, and the environment [1, 3, 10, 19, 20, 23, 24]. These approaches show promise for capturing complex interdependencies and feedback loops within the nexus, though they often face challenges related to data availability and validation.

Our analysis reveals methodological gaps in the current literature. Few studies employ truly integrated approaches that combine quantitative metrics with qualitative insights while accounting for socio-cultural contexts and governance structures. There is limited application of novel data science techniques, such as machine learning and big data analytics, which could help uncover hidden patterns and relationships within the nexus.

### Regions & Diseases Analysis

The regions most studied within the WEFH nexus are South Africa [1, 3, 13], China [21, 22], and the Greater Horn of Africa [19]. China is undergoing rapid urbanization, which affects the food system, water-intensive animal products, and the prevalence of non-communicable diseases (NCDs) [21, 22]. The Greater Horn of Africa challenges overpopulation, climate change, and the COVID-19 pandemic, which have increased water scarcity and food insecurity [19]. South Africa is vulnerable due to its socio-economic and environmental context: being water-stressed with climate change compounding existing socio-economic challenges such as food insecurity and an ailing energy system [1].

The diseases most studied within this nexus are non-communicable diseases (NCDs) such as diabetes, asthma, stroke, cancer [21, 22], and water-borne diseases, such as schistosomiasis and malaria [17, 20]. NCDs are a major concern in urban China: hypertension, diabetes, and stroke affect millions of adults. Water-borne diseases are a significant burden in much of the world, including the Senegal River Basin, where the prevalence of schistosomiasis remains high despite efforts to control it [17].

The COVID-19 pandemic has significantly impacted the WEFH nexus, disrupting supply chains, affecting demand, and altering consumption patterns [18, 20]. The pandemic raised the importance of health in the WEF nexus: individual well-being is closely linked to the availability and quality of water, the energy required for living, and nutritional aspects of food production [2].

### Trends in Literature

Current literature on the WEFH nexus focuses on several trends and areas, reflecting its growing importance in addressing global challenges like climate change, urbanization, and sustainable development. Researchers have started pushing towards integrating health into the WEF nexus, with studies examining how health outcomes are affected by water, energy, and food security [1–3, 17, 24]. Another major trend is the focus on urbanization, with studies examining how urbanization affects the WEF nexus and its impacts on health, livelihoods, and the environment [21, 22], driven by the rapid demand growth for WEF resources in urban areas. A third trend is the focus on climate change, with studies examining how climate change affects the WEF nexus and its impacts on health [1, 3, 13, 18–20, 23, 24]. This recognizes that WEFH nexus can be a valuable framework for adapting to and mitigating climate change. A fourth trend is the focus on sustainable development, with studies examining how the WEF nexus can be used to promote economic development, and social equity [1–3, 12, 13, 15, 19, 22, 24]. This indicates that the WEF nexus as a complex and interconnected system is needed to support sustainable development.

### Health in WEF Literature

Health is investigated to varying extents within the WEF nexus. Some studies explicitly incorporate health as a central component and others treat it as an externality. Our analysis identified four approaches to health integration in the nexus literature:

#### Health as an Outcome

Studies highlighted the direct and indirect impacts of WEF nexus components on human health, including the effects of water quality and sanitation on the prevalence of waterborne diseases [10], the impact of dietary changes and food security on nutrition and non-communicable diseases (NCDs) [21–23], and the effects of energy production and consumption on air pollution and respiratory health [4, 12, 14]. This approach focuses on how resource systems impact health but often fails to capture feedback effects.

#### Health as a Cross-Cutting Dimension

More advanced studies integrate health into the WEF nexus framework, recognizing interdependencies between health and other nexus components [1–3, 13, 19, 24]. This integration acknowledges the crucial role of health in achieving sustainable development goals and emphasizes the need for a holistic approach that considers the complex interactions between health, water, energy, and food security.

#### Health as a Central Nexus Component

The most comprehensive approach positions health as a full and equal component within the nexus, with integrated relationships to water, energy, and food systems. While this approach remains less common in the literature, emerging work by Nuwayhid & Mohtar (2022) and Mutanga et al., (2024) demonstrates its potential for developing truly integrated policies that simultaneously address resource sustainability and human wellbeing [1, 2]. These studies emphasize health not as an afterthought or consequence, but as a foundational element that influences and is influenced by resource systems.

Despite growing recognition of health’s importance in the WEF nexus, our analysis reveals that many studies still treat health as an outcome or externality rather than a central component. This gap stems from the complexity of integrating health into WEF assessments and the lack of established methodologies and metrics for quantifying health-resource interactions. Addressing this limitation represents a key opportunity for advancing WEFH research and practice.

### Analysis of Literature Gaps

Despite advancements in WEFH nexus research, significant methodological gaps persist. Many studies rely on sectoral models that fail to capture the dynamic interdependencies among water, energy, food, and health systems. The application of integrated frameworks such as system dynamics modeling remains limited due to high data requirements and computational complexities. Additionally, the lack of standardized indicators and metrics for health outcomes within the nexus hinders comparative analyses across regions and sectors [3].

Research on the WEFH nexus is disproportionately concentrated in high-income countries, with limited representation of low- and middle-income regions where resource insecurities and health disparities are most pronounced. While studies have extensively examined water and energy interactions in Europe and North America, regions such as Sub-Saharan Africa and South Asia remain underrepresented despite vulnerability to climate change and resource scarcity [5].

In underrepresented regions, data scarcity further exacerbates the challenge of conducting nexus research. Limited access to reliable datasets on water quality, energy consumption, and health outcomes constrains the ability to develop context-specific solutions. Addressing these geographic disparities requires targeted investments in data collection, capacity building, and collaborative research networks that prioritize vulnerable regions [19].

Health remains the least integrated dimension within the WEFH nexus. While the interconnections between water, energy, and food systems are well-documented, their direct and indirect impacts on health are often overlooked or superficially addressed. For example, studies frequently highlight the role of water contamination in disease prevalence but rarely consider broader health indicators, like NCDs linked to resource management practices [18], or the interaction between resource nexus in a goal of maintaining societal health.

To fully integrate health into the nexus, researchers must adopt interdisciplinary approaches that bridge the gap between environmental science, public health, and socio-economic research. This includes developing health-specific metrics and disease burden indices and incorporating them into existing nexus models. Participatory approaches that engage local communities and healthcare stakeholders can ensure health considerations are prioritized in resource management decisions [2].

### Policy and Governance Challenges

Governance frameworks for WEFH nexus are often fragmented, with responsibilities divided among multiple sectors and institutions. Our review identified three critical governance challenges that hinder effective WEFH integration.

#### Institutional Fragmentation

Siloed approaches across water, energy, food, and health ministries/departments create coordination barriers and conflicting policies [28]. Agricultural subsidies meant to enhance food security may promote water-intensive crops in water-scarce regions, thus undermining both water security and public health outcomes.

#### Cross-Scale Governance Gaps

Misalignment between national policies and local implementation creates effectiveness gaps. Many WEFH interventions require coordinated action across governance levels, yet mechanisms for vertical integration often remain weak.

#### Limited Stakeholder Engagement

Technical and top-down approaches often fail to incorporate community perspectives and traditional knowledge [29]. Successful WEFH integration requires participatory governance that engages diverse stakeholders, including marginalized communities affected by resource insecurities.

Participatory governance mechanisms involving stakeholders from diverse sectors and communities can enhance legitimacy and effectiveness of nexus-based policies [13]. This includes fostering inter-agency collaboration, promoting multi-level governance, and incorporating equity considerations into decision-making processes.

## Conceptual Framework of the Water-Energy-Food-Health Nexus

### The Need for a Holistic WEFH Nexus Model

Current research on WEF nexus has made significant progress in understanding resource interdependencies [6, 30, 31], but demands linking these interdependencies with their effects on health. Existing methodologies primarily overlook or treat health as a secondary consequence rather than an integrated factor within resource systems. This gap is evident in the literature, where most studies focus on optimizing water, energy, and food systems without accounting for the feedback loops between resource availability and public health outcomes.

While some studies highlight the health implications of specific resource issues, poor water quality [32–36], food insecurity [37–40], or climate change [41–43], they typically do so in isolation without a systems-based approach. This fragmented perspective fails to capture the interconnected nature of health within the nexus, limiting our ability to develop interventions that simultaneously address resource sustainability and human wellbeing.

The absence of a comprehensive WEFH model presents a critical challenge in regions facing compounded resource insecurities and health inequities. Water scarcity not only limits agricultural production but also increases prevalence of waterborne diseases [44]. Similarly, poor nutrition weakens immunity and exacerbates disease burdens [45].

Health considerations have driven significant resource system interventions. Finland’s nationwide effort to address high heart attack rates in the 1970s led to food system transformations that reduced sodium intake by approximately 30%, resulting in an 84% decline in coronary heart disease mortality [46]. Similarly, introduction of orange-fleshed sweet potato in Mozambique and Uganda reduced vitamin A deficiency among children and mothers [47], while India’s provision of liquefied petroleum gas stoves to over 80 million families improved respiratory health outcomes [48].

These examples demonstrate the bidirectional relationship between health and resource systems: public health trends influence how societies manage water, energy, and food resources, while changes in these systems affect health outcomes. Without a framework that accounts for these multidimensional interactions, decision-making remains reactive rather than proactive, leading to suboptimal policies that fail to address root causes.

**Fig. 5:**
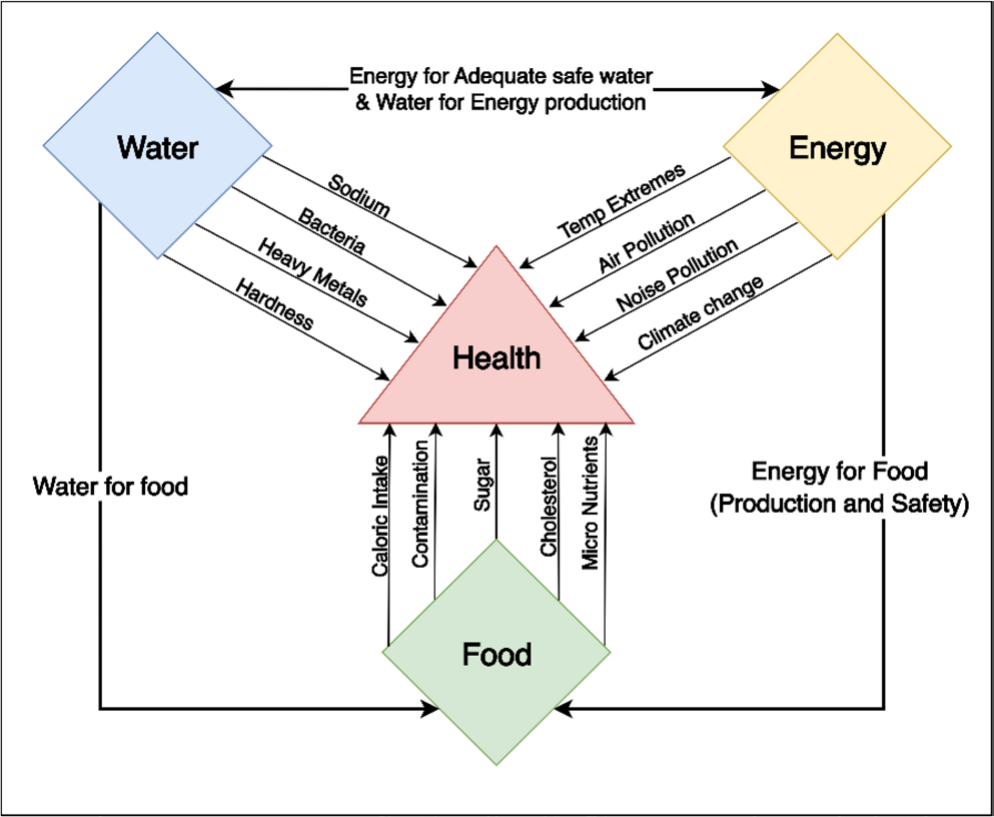
WEFH nexus conceptual framework

To address these shortcomings, we propose a novel WEFH nexus framework that explicitly integrates health as both an outcome and a driver of resource dynamics. This approach moves beyond treating health as a secondary concern to positioning it as a central component with dynamic relationships to water, energy, and food systems (Fig. 5). By identifying specific pathways through which resource dynamics affect health outcomes and how health status influences resource demand and management, this framework enables a more comprehensive understanding of system interactions.

### Health at the Core of the Nexus

Recent global shocks, such as the COVID-19 pandemic, exposed the fragility of global and local resource systems [20]. Lockdowns disrupted food supply chains, energy-dependent healthcare infrastructure, and clean water access that affected vulnerable populations. Such crises underscore the need for a WEFH lens to evaluate resilience and equity. Despite this, scholarly efforts remain uneven. While certain regions (e.g., Sub-Saharan Africa, South Asia) and diseases (e.g., diarrheal illnesses, malnutrition) receive attention due to their acute resource-related health burdens, other critical areas, such as non-communicable diseases linked to resource quantity and quality, remain underexplored.

#### Water-Health Nexus

Water-health nexus involves the interaction between water systems and public health outcomes, emphasizing the critical roles of water availability, quality, and accessibility in shaping disease burdens and community well-being. Key indicators (water quantity, contamination levels, accessibility metrics) serve as vital measures of systemic resilience. In 2022, 73% of the global population (roughly 6 billion people) used a safely managed drinking water service [49]. This yet leaves approximately 2.2 billion people without safely managed water, contributing to 829,000 annual deaths from diarrheal diseases linked to poor water quality [50].

Water quality is as important as quantity for health. In 2022, at least 1.7 billion people used drinking water sources contaminated with fecal matter [49]. Chemical contaminants like heavy metals and pesticides also threaten health globally. Arsenic exposure caused skin lesions, cardiovascular disease, and cancers in populations across South Asia and Latin America [51], while water quality monitoring remains inadequate in many countries, with over 40% of surveyed water bodies worldwide classified as severely polluted [49].

Table 3 summarizes water related attributes and their health impacts. Contaminants can lead to severe illnesses including diarrhea, cancer, liver disease, and kidney stones. High sodium levels contribute to hypertension and cardiovascular disease; inadequate water intake can cause dehydration, fatigue, and kidney stones.

**Table 3:** Main health indicators attributed to water

Resource Indicator	Main Attributes	Health Indicators	Reference
Water Quality	Sodium	Cardiovascular Diseases, Hypertension, Kidney stones	[52–54]
	Microbial Contamination	Diarrhea, Vomiting	[55, 56]
	Nitrates and Nitrites	Cancers, Methemoglobinemia	[57, 58]
	Heavy Metals	Cancers	[59]
	Disinfection Byproducts	Liver, Cancers	[60, 61]
Water Quantity	Water intake	Hydration, Fatigue, Kidney stones	[62, 63]
	Hygiene	Feco-oral contamination, Skin diseases	[64, 65]

#### Food-Health Nexus

Global food availability has improved over decades, yet in 2022 an estimated 735 million people (9.2% of the world population) faced chronic undernourishment [66]. On the other hand, adult obesity rates have more than doubled globally from 7% in 1990 to around 16% by 2022 [67]. Poor diets and excess weight are key drivers of diet-related NCDs like heart disease and diabetes [68]. Table 4 summarizes the key interactions in this nexus. Dietary factors, nutritional deficiencies, and food safety present health risks leading to acute illnesses and chronic conditions.

**Table 4:** Main health indicators attributed to food

Resource Indicator	Main Attributes	Health Indicators	Reference
Food Quality	Sodium	Cardiovascular Diseases, Hypertension, Kidney stones	[52–54]
	Sugar	Obesity, Diabetes	[68, 69]
	Proteins	Muscle wasting, Malnutrition	[70, 71]
	Fats	Obesity, Cardiovascular disease	[72, 73]
	Iron	Anemia, Fatigue	[74, 75]
	Calcium	Osteoporosis	[76]
	Vitamin D	Osteomalacia	[77]
	Vitamin A	Immune system function, Vison	[78, 79]
	Microbial Contamination	Diarrhea, Vomiting	[56, 80]
	Heavy Metals	Cancers	[59]
Food Quantity	Caloric Intake	Malnutrition	[81]

#### Energy-Health Nexus

Energy health examines the dual role of energy systems as drivers of water and food systems and as sources of environmental pollution. Access to modern energy is a fundamental determinant of health and an explicit focus of SDG 7 (affordable and clean energy for all). Significant progress has been made in expanding electricity access globally, yet basic energy poverty persists. As of 2021, an estimated 675 million people worldwide live without electricity, and 2.3 billion, nearly one-third of humanity, lack access to clean fuels and technologies for cooking [82]. Furthermore, energy access is highly uneven across regions, exacerbating health inequities. The vast majority of people without electricity live in low- and lower-middle-income countries, particularly sub-Saharan Africa, which accounts for about 75–80% of the global population lacking power [82].

The lack of electricity constrains living conditions (no refrigeration, hindered access to safe water). Meanwhile, household air pollution represents a significant intersection of the energy-health nexus globally. Approximately 2.1 billion people, around one-third of the global population, rely on inefficient cooking methods using fuels such as biomass, coal, and kerosene, leading to harmful indoor air pollution [83]. This exposure results in approximately 3.2 million premature deaths annually, including over 237,000 children under five, mainly from noncommunicable diseases like stroke, ischemic heart disease, and lung cancer [84].

Table 5 presents the main energy attributable health impacts reported in literature. As discussed previously, air pollution from indoor and outdoor sources is linked to respiratory diseases, lung cancer, heart disease, and climate change-related health risks. Noise pollution contributes to sleep disturbances, stress, and hearing loss. Extreme heat exposure can lead to dehydration and exacerbate cardiovascular diseases; cold exposure increases risk of hypothermia and respiratory infection.

**Table 5:** Main health indicators attributed to energy

Resource Indicator	Main Attributes	Health Indicators	Reference
Energy Quality	Air Pollution (indoor/ outdoor)	Respiratory disease, heart disease, lung cancer, Asthma, CO poisoning, Climate Change	[85–88]
	Noise Pollution	Sleep disturbances, Stress	[89]
Energy Availability	Extreme Heat Exposure	dehydration, exacerbation of cardiovascular diseases	[90, 91]
	Extreme Cold Exposure	Hypothermia, respiratory infections	[92, 93]

Integrating health into the nexus framework ensures that the impact of resource management decisions on human well-being are comprehensively addressed. This is not easily done, despite its clear relevance. Health scientists are not trained to engage the nexus in health care or population research. From decades of research, there is an abundance of literature about water quality and quantity on health [51], but not in the context of low energy access or in relation to low food access that leads to lower immunity and more severe complications and consequences of waterborne diseases. Engineers and non-health scientists are also not trained to understand the complex health risk factors and the multifactorial nature of most diseases, especially chronic noncommunicable diseases. While engineering and health sciences are applied, their approaches to addressing the nexus are very different, making it challenging to plan interdisciplinary sciences. However, with the advancement of science, the major problems facing the planet and people such as climate change, the interdisciplinary approach of the WEFH is a necessity. The Global Center for Climate Change and Water Energy Food and Health systems (https://hwsph.ucsd.edu/research/programs-groups/gcccwefh.html) is one example of the success of this interdisciplinary approach: engineers, health scientists, social scientists, data scientists, and other disciplines work together to learn the approaches and perspectives of each other to advance solutions. As this review demonstrates, there is a growing number of publications in literature considering health in the nexus. This is mostly led by engineers and physical scientists; health scientists, engineers, and practitioners will need to develop the skills of team science and understand how to work with experts in the other areas of the nexus, providing insight and expertise in health to the nexus. By embedding health as a critical component, the WEFH nexus provides a pathway to equitable and sustainable development, particularly in regions where health inequities intersect with resource insecurities.

## Conclusion

The integration of health into the Water-Energy-Food (WEF) nexus represents a necessary paradigm shift in resource management and policy development. This review reveals that while water, energy, and food systems are inherently linked to public health, the current nexus literature often treats health as a secondary outcome rather than an integral component. This oversight limits the effectiveness of strategies aimed at ensuring sustainable and equitable resource distribution, particularly in regions facing both resource insecurity and health disparities. Addressing this gap requires a more comprehensive approach that explicitly incorporates health into WEF nexus frameworks.

A major challenge in advancing the Water-Energy-Food-Health (WEFH) nexus is the fragmented governance structures regulating these sectors. Institutional silos and policy misalignment hinder coordinated decision-making, exacerbating trade-offs that negatively impact public health. Methodological barriers, including lack of standardized health indicators and limited cross-sectoral data integration, further restrict the ability to quantify direct and indirect health impacts of resource management decisions. Bridging these gaps requires interdisciplinary collaboration, improved data collection mechanisms, and development of integrated models that capture the dynamic interactions between WEFH components.

The literature review highlights increasing interest in health within the nexus context, particularly in response to global crises such as climate change and pandemics. However, research remains disproportionately concentrated in high-income regions, leaving significant knowledge gaps in low- and middle-income countries where resource insecurities and health burdens are most severe. Future studies should prioritize underrepresented regions and explore how localized challenges can be addressed through context-specific WEFH strategies.

To advance the WEFH nexus, this review presents the complex resource-health interactions which serves as foundational step towards conceptualizing health as an outcome of sustainable resource management. This framework underscores the need for integrated approaches that recognize interdependencies between water quality, energy accessibility, food security, and public health. By embedding health considerations into nexus-based governance and decision-making, resource management strategies can be more effective in promoting resilience, equity, and sustainability.

Ultimately, integrating health into the WEF nexus is critical towards achieving holistic, long-term sustainability. Policymakers, researchers, and practitioners must adopt a systems-thinking approach that recognizes the multifaceted nature of resource-health interactions. Future research should focus on developing interdisciplinary methodologies, comprehensive WEFH quantification, fostering data-sharing initiatives, and implementing governance mechanisms that facilitate cross-sectoral collaboration. By doing so, the WEFH nexus can serve as a strong foundation for addressing some of the most pressing global challenges related to resource security, environmental sustainability, and human well-being.

## Key references

- Nuwayhid I, Mohtar R (2022) The Water, Energy, and Food Nexus: Health is yet Another Resource. Front Environ Sci 10: 10.3389/fenvs.2022.879081
  - ○ The paper highlights the importance of incorporating health into the WEF nexus framework for more comprehensive solutions.
- Javan K, Darestani M, Ibrar I, Pignatta G (2025) Interrelated issues within the Water-Energy-Food nexus with a focus on environmental pollution for sustainable development: A review. Environ Pollut 368:125706. 10.1016/j.envpol.2025.125706
  - ○ Examines environmental pollution within the Water-Energy-Food (WEF) nexus, focusing on sources, interconnectedness, and feedback loops to inform sustainable policy development.
- Calder RSD, Grady C, Jeuland M, Kirchhoff CJ, Hale RL, Muenich RL (2021) COVID-19 Reveals Vulnerabilities of the Food-Energy-Water Nexus to Viral Pandemics. Environ Sci Technol Lett 8:606–615. 10.1021/acs.estlett.1c00291
  - ○ Evaluates how pandemics impact the FEW nexus systems and exposes it to vulnerabilities.

## Data Availability

All data supporting the findings of this study are available through PubMed and Web of Science Databases: [https://pubmed.ncbi.nlm.nih.gov/]; [https://webofscience.com/]
